# The Impact of Active Screening and Management on COVID-19 in Plateau Region of Sichuan, China

**DOI:** 10.3389/fmed.2022.850736

**Published:** 2022-05-10

**Authors:** Jia-jie Lu, Guo-guo Jiang, Xiang He, Kai-ju Xu, Hong Yang, Rui Shi, Ying Chen, Yu-yao Tan, Lang Bai, Hong Tang, Guo-ping Li

**Affiliations:** ^1^Center of Infectious Diseases, West China Hospital of Sichuan University, Chengdu, China; ^2^Department of Hospital Infection Management, Chengdu Second People Hospital, Chengdu, China; ^3^Laboratory of Allergy and Precision Medicine, Chengdu Institute of Respiratory Health, the Third People's Hospital of Chengdu, Affiliated Hospital of Southwest Jiaotong University, Chengdu, China; ^4^Department of Pulmonary and Critical Care Medicine, Chengdu Third People's Hospital Branch of National Clinical Research Center for Respiratory Disease, Affiliated Hospital of ChongQing Medical University, Chengdu, China; ^5^Department of Infectious Diseases, Sichuan Academy of Medical Sciences and Sichuan Provincial People's Hospital, Chengdu, China; ^6^People's Hospital of Ganzi Tibetan Autonomous Prefecture, Ganzi Tibetan Autonomous Prefecture, Kangding, China

**Keywords:** COVID-19, hypoxic plateau area, clinical characteristics, control strategies, severe pneumonia

## Abstract

**Background:**

In December 2019, the cases of pneumonia of unknown etiology emerged in Wuhan, China, and rapidly spread throughout the country. The disease was later designated by the World Health Organization (WHO) as Coronavirus Disease 2019 (COVID-19) caused by severe acute respiratory syndrome coronavirus 2 (SARS CoV-2). Few studies have assessed the clinical characteristics of COVID-19 and control strategies used to mitigate disease spread in high-altitude plateau regions of China.

**Study Objective:**

To assess the impact of real-world strategies to control COVID-19 spread in remote plateau regions.

**Methods:**

A retrospective study was performed to assess the epidemiology of COVID-19 and strategies used to control disease spread in the high-altitude plateau of Sichuan, China from 24 January 2020 to 19 March 2020.

**Results:**

COVID-19 spread and outbreaks in Sichuan were attributed to mass gatherings. A total of 70 patients and 20 asymptomatic individuals were found in the hypoxic plateau region of Sichuan. Twelve patients were admitted after the onset of symptoms, while 58 patients and 20 asymptomatic individuals were found by active screening. The symptomatic patients included those with uncomplicated illness (16/70, 22.9%), mild pneumonia (44/70, 62.9%), and severe pneumonia (10/70, 14.3%). Most patients in the study area showed relatively mild and atypical symptoms such as low or no fever and dyspnea. The incidence of severe pneumonia, fever, dyspnea, and interstitial abnormalities identified by chest CT were all significantly lower in screened patients than those admitted after symptom onset (*P* < 0.05). Severe pneumonia was noted in patients with chronic conditions like hypertension, diabetes etc. as compared to less severe pneumonia in healthy subjects (P <0.05). No patients died and all were eventually discharged.

**Conclusion:**

Mass gatherings increased risk of spread of SARS-CoV-2 responsible for COVID-19. Active screening and early management have collectively contributed to reduced incidence of severe pneumonia and satisfactory prognoses of infections with COVID-19 in this hypoxic plateau region.

## Introduction

In December 2019, a novel coronavirus, now designated SARS-CoV-2, was identified as the cause of a cluster of pneumonia cases in Wuhan, a major city in Hubei Province, China. The disease spread rapidly throughout China, with an increasing number of cases reported globally (1–4). In February 2020, the WHO designated COVID-19 a global health emergency and upgraded the disease to pandemic status in early March 2020 (5). As of July 25, 2020, more than 15 million cases of COVID-19 have been reported to WHO (6). Of these, more than 80,000 cases were from China, the majority of whom were from Hubei and the surrounding provinces (6).

Previous studies have described the clinical characteristics of infected patients in and outside of Wuhan, contributing to an understanding of COVID-19-related epidemiological, clinical, laboratory, and radiological features as well as treatment outcomes (7–9). The pathological characteristics of COVID-19 include lung interstitial mononuclear inflammatory infiltrates and diffuse alveolar damage with cellular fibromyxoid exudates (10). Many patients presented with organ function damage and required mechanical ventilation. Older patients (>65 years of age) with comorbidities and Acute Respiratory Distress Syndrome (ARDS) are at increased risk of death (11). According to a report from the Chinese Center for Disease Control and Prevention, as of 11 February 2020, 1,023 deaths occurred among the 44,672 confirmed cases, with the overall case fatality rate of 2.3 percent (12).

**Figure 1:**
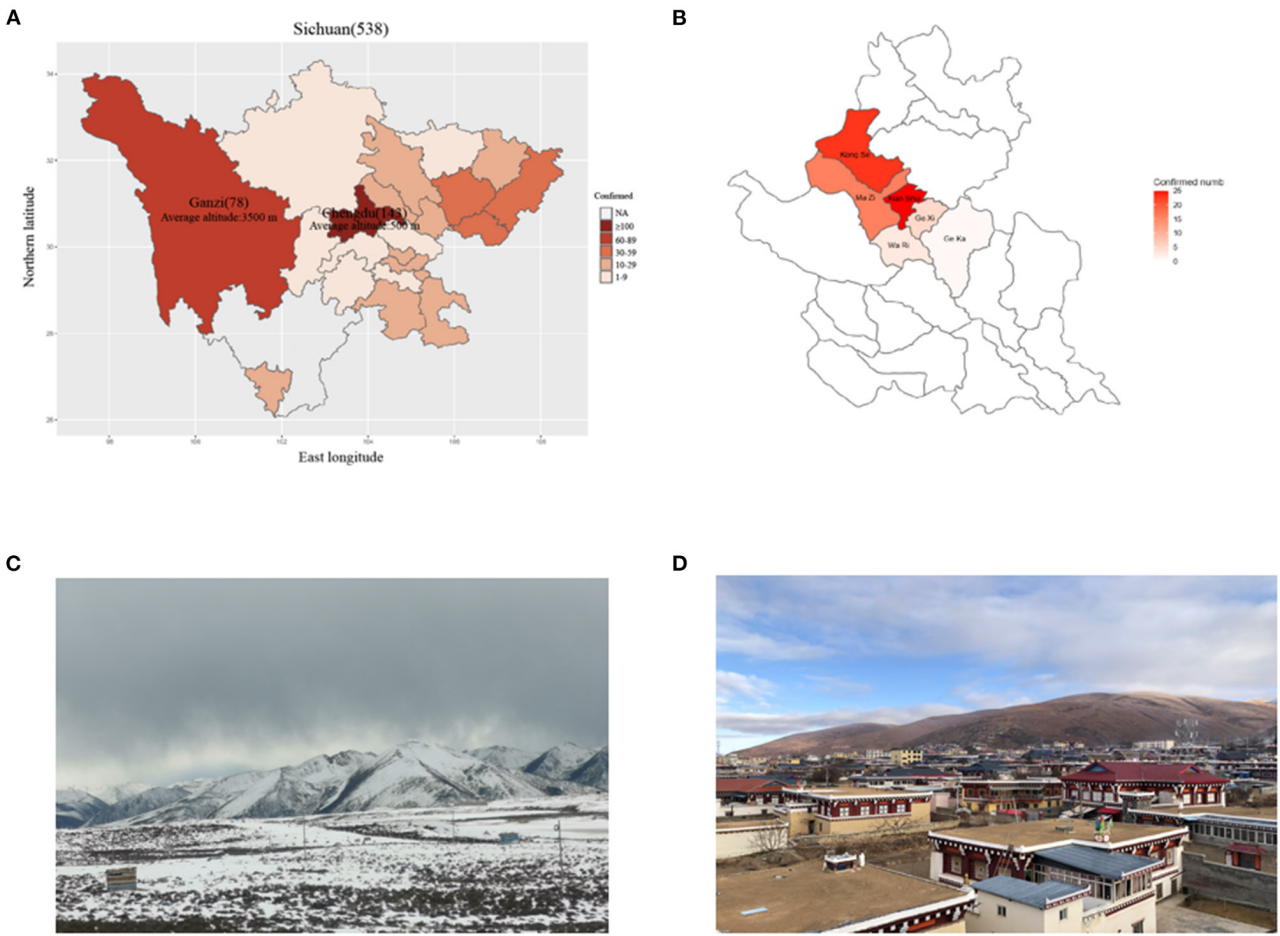
The distribution of COVID-19 cases across Sichuan province. **(A)** The distribution of patients across Sichuan province. **(B)** The distribution of patients across Daofu County. **(C)** The geographical features of Ganzi Tibetan Autonomous Prefecture. **(D)** High crowd density in Daofu County. The official statistics of all documented laboratory-confirmed COVID-19 cases throughout Sichuan province (March 19, 2020).

In late January 2020, an outbreak of COVID-19 was reported in Daofu, a county in the Ganzi Tibetan Autonomous Prefecture of Sichuan Province, China (Figures 1A,B). The area lies at the margin of the Qinghai-Tibet plateau, surrounded by perennially snow-topped mountains. Daofu has an average altitude of 3,245 meters (range 2,670–5,820 meters), covers an area of 7,053 square kilometers, and has a population of more than 55,000. The barometric pressure at this altitude ranges from ~72.40 to 46.61 kPa compared to 101.33 kPa at sea level. It is a cold, dry, and oxygen-depleted region in winter. The oxygen content of the air at these altitudes ranges from 10 to 15%, compared to 21% at sea level, and there is a significant day/night temperature differential (Figure 1C), most people in Daofu county live in relatively concentrated areas (Figure 1D). Tibetans have lived at very high altitudes for thousands of years, and have adapted to these regions using complex hypoxia-response pathways (13). Only a few reports detailing the clinical features of COVID-19 in highland areas of China have been published (14, 15).

While most transmission events occurred during the pre-symptomatic phase (59.2%), transmissibility was not significantly different between symptomatic and asymptomatic individuals in Wuhan (8, 9). Available data suggests that at least one-third of SARS-CoV-2 infections are asymptomatic (16), and nearly three-quarters of individuals who tested positive but had no symptoms at the time of testing remain asymptomatic (17). Thus, control strategies for COVID-19 should consider the prevalence and transmission risk of individuals with asymptomatic SARS-CoV-2 infection.

The aim of the present study is to describe the clinical features of COVID-19 patients from high-altitude regions and investigate the potential strategies used to control the spread and decrease the severity of this disease.

## Methods

### Data Sources

A retrospective study was conducted to assess the clinical characteristics of 70 symptomatic patients with COVID-19 in Daofu, China from 24 January 2020 to 19 March 2020. Symptomatic cases were diagnosed using WHO interim guidance (18). In addition, 20 asymptomatic infections were found by active screening. Asymptomatic infection was defined as the detection of SARS-CoV-2 infection in a patient lacking symptoms and radiologic abnormalities (17). The Institutional Review Board Committee of West China Hospital of Sichuan University approved the study protocol. Informed consent was obtained from all cases. The study conformed to the ethical guidelines of the 1975 Declaration of Helsinki (19).

Data were collected on 70 symptomatic patients with COVID-19 admitted to a designated hospital in Daofu. Exposure history during the 2 weeks before illness onset was recorded, including the dates and times of close contacts (gathering, living, or working together) with individuals who had confirmed or suspected SARS-CoV-2 infection. The incubation period was defined as the duration from contact with the transmission source to the onset of symptoms (20). Clinical records, laboratory, and chest computed tomography (CT) findings were obtained from the electronic medical records of all patients with COVID-19 who were reported by the local health authority. Two researchers independently reviewed the data collection forms to verify the data. The researchers also directly communicated with patients or their families to obtain epidemiological and symptom data which were not available from electronic medical records.

### Laboratory Confirmation and Treatment

Investigations included a complete blood count, coagulation profile, serum biochemistry (including renal and liver function, creatine kinase, lactate dehydrogenase, and electrolytes), and chest CT (Optima CT520). Respiratory specimens, including sputum or pharyngeal swabs collected from all patients at admission, were tested using real-time polymerase chain reaction (RT-PCR) specific for SARS-Cov-2 RNA.

Most patients received antiviral treatment along with traditional Chinese medicine, including lopinavir and ritonavir (400 mg twice daily and 100 mg twice daily, respectively), ribavirin (intravenously 500 mg twice daily), or arbidol (200 mg three times daily). Patients received treatment with corticosteroids (40–80 mg/day) for 3–5 days when their resting respiratory rate was >30 per minute, oxygen saturation was <93% or multiple pulmonary lobes showed >50% disease progression in 48 h. Oxygen support (e.g., nasal cannula and mask) was administered to patients based on the severity of the hypoxemia. Since COVID-19 cases first appeared during influenza season, antibiotics (oral and intravenous) and oseltamivir (orally 75 mg twice daily) were empirically administered. Repeat SARS-CoV-2 testing was done for patients suspected of having COVID-19 to show viral clearance before hospital discharge or discontinuation of isolation.

### Statistical Analysis

Data were reported as the mean ± standard deviation for normal continuous variables and the median (interquartile range) for non-normal continuous variables, while the frequency was used for discrete variables. In the univariate analysis, the Student's *t*-test and ANOVA with Bonferroni adjustments were used for continuous samples, and the Fisher's exact or Chi-square tests were used for qualitative samples. Non-parametric alternatives (Mann-Whitney U and Kruskal-Wallis tests) were used for non-normal distributions. Categorical variables were summarized as the counts and percentages in each category. Patients were grouped into those with uncomplicated illness, mild pneumonia, and severe pneumonia based on WHO interim guidance (13). Distribution maps of COVID-19 were produced using the *nCov2019* R package. All analyses were performed using SPSS (version 22.0) and R software (version 3.5).

## Results

### Epidemiology and Control Strategies

**Figure 2:**
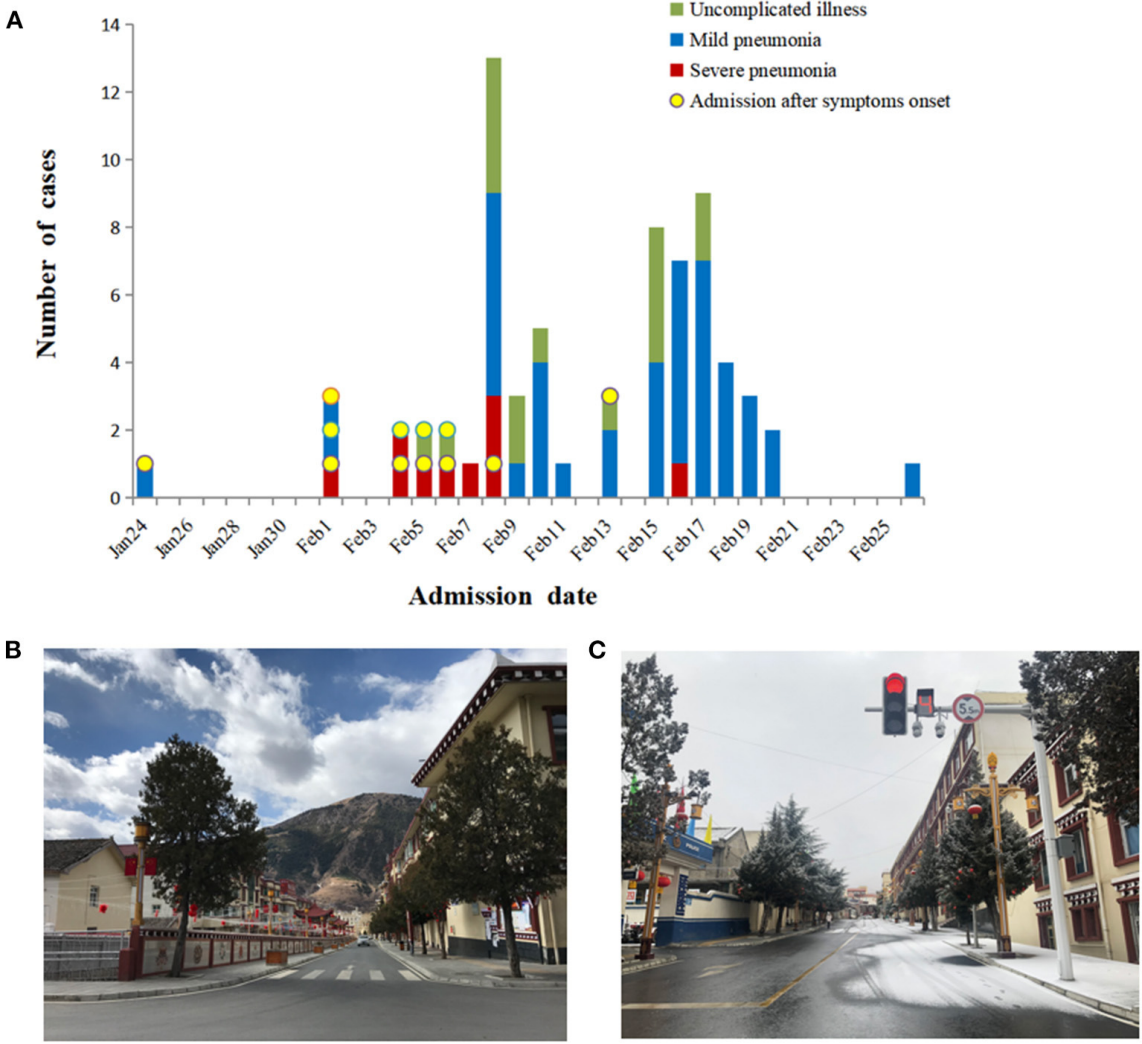
Timeline of COVID-19 cases after onset of illness or active screening and disease control strategies. **(A)** Timeline of COVID-19 cases after onset of illness or active screening. **(B,C)** Disease control strategies led to empty streets and shopping centers.

A total of 70 patients and 20 individuals with asymptomatic infections were found in Daofu. The first patient identified with COVID-19 had returned to Daofu from Chengdu city on 18 January, 2020, and had not traveled to or been in contact with individuals from Wuhan. Early in the outbreak, most patients were involved in weddings, funerals, or familial gatherings in Daofu and were admitted after the onset of symptoms. Notably, the 12 patients admitted after symptom onset included those with uncomplicated illness (3/12, 25.0%), mild pneumonia (3/12, 25.0%) and severe pneumonia (6/12, 50.0%) (Figure 2A).

**Figure 3:**
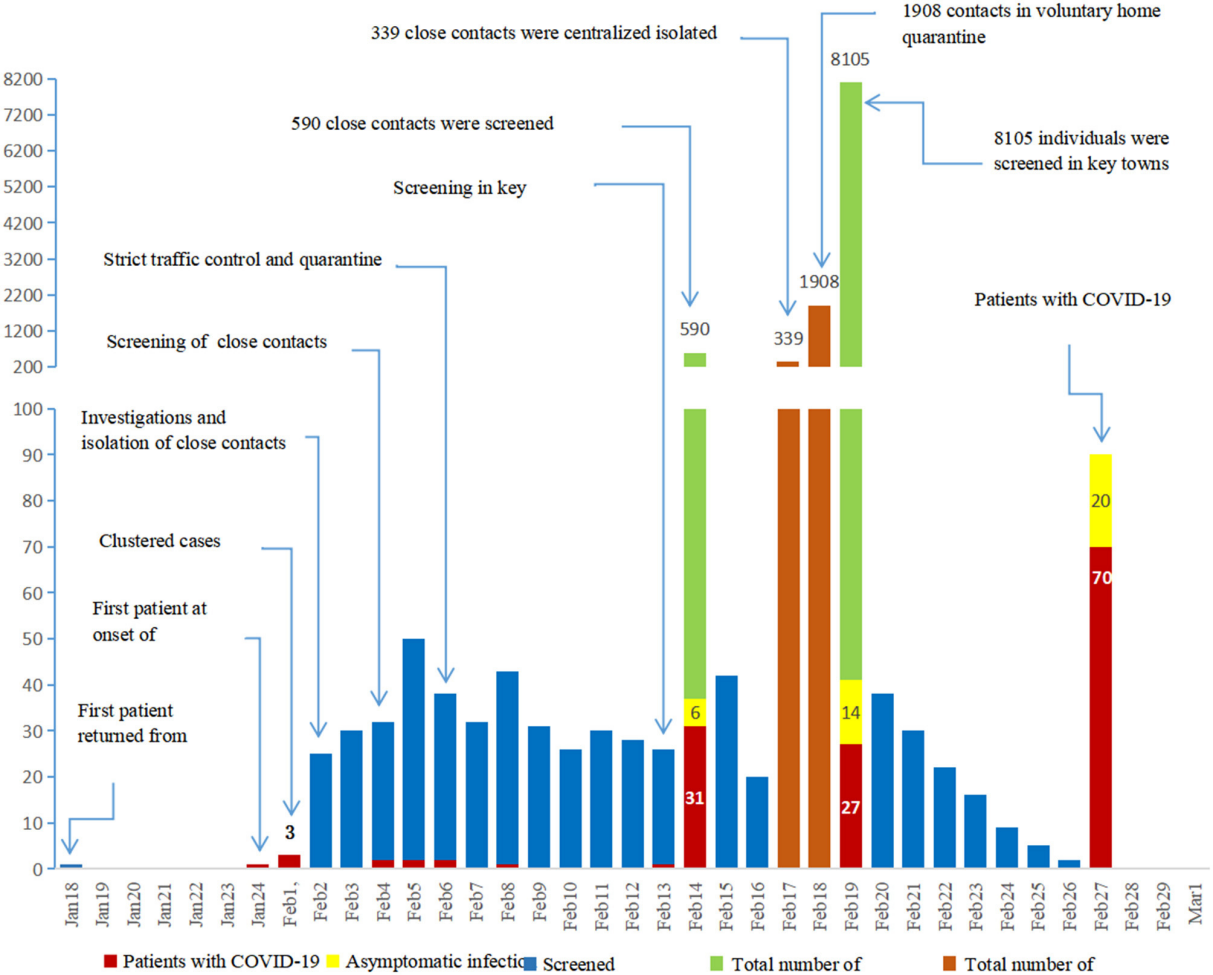
Since the outbreak of COVID-19, isolation, quarantining and active screening have been implemented. In total, 8695 close contacts were actively screened with RT-PCR.

The COVID-19 outbreak in China led to the implementation of strict isolation, quarantining, and active screening procedures (Figures 2B,C). A total of 339 close contacts were placed in centralized isolation and 1,908 were placed in voluntary home isolation in Daofu. Active screening of 590 close contacts began on 4 February 2020, and SARS-CoV-2 nucleic acid was detected in 37 cases. Soon after, 8,105 individuals were screened in key towns with clustered cases and 41 COVID-19 cases were confirmed using RT-PCR. As of 1 March 2020, 8,695 individuals were actively screened for COVID-19. Of 78 individuals who tested positive for COVID-19, 20 were asymptomatic and 58 symptomatic patients were found by active screening (Figure 3).

### Demographic and Clinical Features of the Symptomatic Patients

According to WHO interim guidance, 58 symptomatic patients who were detected by active screening included those with uncomplicated illness (13/58, 22.4%), mild pneumonia (41/58, 70.7%), and severe pneumonia (4/58, 6.9%). In contrast, 12 patients admitted after symptoms onset included patients with uncomplicated illness (3/12, 25.0%), mild pneumonia (3/12, 25.0%), and severe pneumonia (6/12, 50.0%). The incidence of severe pneumonia was significantly lower in screened patients (4/58, 6.9%) than those who went to the hospital after symptom onset (6/12, 50.0%) (*P* < 0.05). The incidence of fever, dyspnea, and chest CT interstitial abnormalities were also lower among screened patients (all *P* < 0.05) (Table 1).

**Table 1:** Contrast between active screening and passive admission to hospital.

	**Admission after symptoms onset *N* = 12**	**Active screening *N* = 58**	***p*-value**
COVID-19 -*n*/*N* (%)			<0.001
Uncomplicated illness	3/12 (25.0)	13/58 (22.4)	
Mild pneumonia	3/12 (25.0)	41/58 (70.7)	-
Severe pneumonia	6/12 (50.0)	4/58 (6.9)	-
Signs and symptoms -*n*/*N* (%)			
Fever	4/12 (33.3)	5/58 (8.6)	0.041
Cough	7/12 (58.3)	46/58 (79.3)	0.281
Fatigue	6/12 (50.0)	36/58 (62.1)	0.542
Dyspnoea	5/12 (41.7)	4/58 (6.9)	0.006
Abnormalities on chest CT -*n*/*N* (%)			
Ground-glass opacity	0/12 (0.0)	13/57 (22.8)	0.104
Local patchy shadowing	0/12 (0.0)	5/57 (8.8)	0.578
Bilateral patchy shadowing	6/12 (50.0)	24/57 (42.1)	0.751
Interstitial abnormalities	3/12 (25.0)	1/57 (1.8)	0.015

*Data are presented as n/N (%)*.

*p-values comparing severe pneumonia and the other cases are from Fisher's exact test*.

Of the 70 symptomatic patients, 68 were Tibetan, and 2 were Han. More than half of the 70 patients (38, 54.3%) were male. The median age was 45 years (IQR 28–54 years) and ranged from 3 to 77 years. Thirty (42.9%) patients were 18–45 years of age, including one pregnant woman, 27 (38.6%) were 46–65 years of age, 5 (7.1%) were <18 years of age, and 8 (11.4%) were >65 years of age. None of the patients had traveled to or been in contact with individuals from Wuhan. Forty-two (60.0%) of the patients were associated with clusters of whom 14 (20.0%) were linked to familial clusters.

Thirty of the 70 patients (42.9%) had at least one underlying disease such as hypertension or chronic obstructive pulmonary disease. The most common symptoms were cough (53, 75.7%) and fatigue (42, 60.0%) and less common symptoms included fever (9, 12.9%), expectoration (9, 12.9%), dyspnea (9, 12.9%), headache (4, 5.7%), myalgia (4, 5.7%), diarrhea (4, 5.7%), nausea or vomiting (4, 5.7%), abdominal pain (3, 4.3%), chest pain (1, 1.4%), and sore throat (1, 1.4%). Patients were grouped into those with uncomplicated illness (16/70, 22.9%), mild pneumonia (44/70, 62.9%) and severe pneumonia (10/70, 14.3%). The average age differed significantly between these groups (*P* < 0.001) and underlying disease was more common among cases with severe pneumonia than among non-severe cases (including those with uncomplicated illness or mild pneumonia) (*P* < 0.05). The incidence of fever, expectoration, dyspnea and headache differed between the groups (all *P* < 0.05). In particular, patients with severe pneumonia had a higher incidence of dyspnea than non-severe cases (*P* < 0.001) (Table 2).

**Table 2:** Clinical characteristics of 70 patients with COVID-19.

**Clinical characteristics, symptoms or signs**	**All patients (*N* = 70)**	**Disease severity**			***p*-value**
		**Uncomplicated illness (*N* = 16)**	**Mild pneumonia (*N* = 44)**	**Severe pneumonia (*N* = 10)**	
**Characteristics**					
Age, median (range) -years	45 (28–54)	19 (12–28)	49 (37–55)	52 (45–57)	<0.001
Age group—*n* (%)					<0.001
<18	5 (7.1)	5 (31.3)	0 (0.0)	0 (0.0)	
18–45	30 (42.9)	9 (56.3)	19 (43.2)	2 (20.0)	–
46–65	27 (38.6)	2 (12.5)	18 (40.9)	7 (70.0)	–
>65	8 (11.4)	0 (0.0)	7 (15.9)	1 (10.0)	–
Sex—*n* (%)					0.858
Women	32 (45.7)	8 (50.0)	19 (43.2)	5 (50.0)	
Men	38 (54.3)	8 (50.0)	25 (56.8)	5 (50.0)	–
Nationality—*n* (%)					0.019
Han	2 (2.9)	0 (0.0)	0 (0.0)	2 (20.0)	
Tibetan	68 (97.1)	16 (100.0)	44 (100.0)	8 (80.0)	–
Underlying diseases—*n* (%)	30 (42.9)	4 (25.0)	18 (40.9)	8 (80.0)	0.011
Hypertension	17 (24.3)	1 (6.2)	12 (27.3)	4 (40.0)	0.093
Digestive system disease	8 (11.4)	2 (12.5)	4 (9.1)	2 (20.0)	0.489
Tuberculosis	7 (10.0)	2 (12.5)	5 (11.4)	0 (0.0)	0.719
Cardiopathy	4 (5.7)	0 (0.0)	2 (4.5)	2 (20.0)	0.130
Chronic obstructive pulmonary disease	3 (4.3)	0 (0.0)	2 (4.5)	1 (10.0)	0.482
Diabetes	3 (4.3)	0 (0.00)	0 (0.0)	3 (30.0)	0.002
Hepatitis B infection	3 (4.3)	2 (12.5)	1 (2.3)	0 (0.0)	0.180
Signs and symptoms—*n* (%)					
Fever	9 (12.9)	1 (6.2)	4 (9.1)	4 (40.0)	0.029
Cough	53 (75.7)	12 (75.0)	32 (72.7)	9 (90.0)	0.782
Fatigue	42 (60.0)	8 (50.0)	27 (61.4)	7 (70.0)	0.561
Expectoration	9 (12.9)	1 (6.2)	4 (9.1)	4 (40.0)	0.029
Dyspnoea	9 (12.9)	1 (6.2)	2 (4.5)	6 (60.0)	<0.001
Headache	4 (5.7)	1 (6.2)	0 (0.0)	3 (30.0)	0.004
Myalgia	4 (5.7)	0 (0.0)	3 (6.8)	1 (10.0)	0.456
Diarrhoea	4 (5.7)	1 (6.2)	2 (4.5)	1 (10.0)	0.769
Nausea or vomitting	4 (5.7)	0 (0.0)	1 (2.3)	2 (20.0)	0.084
Abdominal pain	3 (4.3)	0 (0.0)	2 (4.5)	1 (10.0)	0.482
Chest pain	1 (1.4)	0 (0.0)	1 (2.3)	0 (0.0)	1.000
Sore throat	1 (1.4)	0 (0.0)	1 (2.3)	0 (0.0)	0.782
Heart rate–Times/min	86 (77–96)	87 (79–98)	85 (74–94)	92 (82–98)	0.838
Oxyhemoglobin saturation—%	92 (90-95)	92 (91–95)	93 (90–96)	88 (85–90)	0.001

*Data are presented as medians (interquartile ranges, IQR) and n (%)*.

*p-values comparing severe pneumonia and the other cases are from χ^2^ test, Fisher's exact test, or Kruskal-Wallis H test*.

Laboratory and radiographic results are shown in Table 3. A percentage of patients had leucopenia (7/70, 10.0%) or lymphopenia (24/70, 34.3%) on admission, and elevated C-reactive protein was observed in 40.3% of patients. Elevated alanine aminotransferase, aspartate aminotransferase, glutamyl transpeptidase, and lactate dehydrogenase levels were also common. Patients with severe pneumonia had more prominent laboratory abnormalities, including lymphopenia, elevated C-reactive protein levels, and elevated lactate dehydrogenase levels than non-severe cases (all *P* < 0.05). On admission, all patients with pneumonia had normal serum procalcitonin levels.

**Table 3:** Laboratory and radiographic findings of 70 patients with COVID-19.

**Laboratory and radiographic findings**	**All patients (*n* = 70)**	**Disease severity**			***p*-value**
		**Uncomplicated illness (*n* = 16)**	**Mild pneumonia (*n* = 44)**	**Severe pneumonia (*n* = 10)**	
White blood cell count, ×10^9^/L	5.3 (4.8–6.6)	5.6 (5.2–6.8)	5.4 (4.5–6.4)	5.2 (3.9–5.9)	0.380
<4	7/70 (10.0)	1/16 (6.2)	4/44 (9.1)	2/10 (20.0)	0.516
>10	3/70 (4.3)	1/16 (6.2)	1/44 (2.3)	1/10 (10.0)	0.309
Neutrophil count, ×10^9^/L	3.7 (2.9–5.1)	3.9 (2.7–2.9)	3.6 (3.0–5.2)	3.4 (2.7–5.4)	0.850
Lymphocyte count, ×10^9^/L	1.3 (0.9–1.6)	1.7 (1.3–2.2)	1.3 (0.9–1.5)	0.9 (0.5–1.7)	0.006
<1.1	24/70 (34.3)	2/16 (12.5)	15/44 (34.1)	7/10 (70.0)	0.011
Haemoglobin, g/dL	146 (125–161)	134 (122–154)	149 (129–161)	159 (132–168)	0.380
Platelet count, ×10^9^/L	131 (100–176)	176 (98–221)	131 (102–174)	107 (84–130)	0.162
<100	18/69 (26.1)	4/16 (25.0)	10/43 (23.3)	4/10 (40.0)	0.617
Prothrombin time, s	12.7 (11.9–13.6)	12.9 (12.3–13.7)	12.6 (11.9–13.3)	13.8 (11.5–15.0)	0.620
Fibrinogen, g/L	2.5 (2.1–3.1)	2.2 (1.9–2.8)	2.5 (2.1–3.0)	3.2 (2.1–4.2)	0.179
Alanine aminotransferase >40 U/L—*n*/*N* (%)	32/70 (45.7)	5/16 (31.2)	22/44 (50.0)	5/10 (50.0)	0.417
Aspartate aminotransferase>40 U/L—*n*/*N* (%)	22/70 (31.4)	1/16 (6.2)	16/44 (36.4)	5/10 (50.0)	0.022
Alkaline phosphatase>150 U/L—*n*/*N* (%)	12/70 (17.1)	7/16 (43.8)	5/44 (11.4)	0/10 (0.0)	0.006
Glutamyl transpeptidase >50 U/L—*n*/*N* (%)	32/70 (45.7)	1/16 (6.2)	26/44 (59.1)	5/10 (50.0)	0.001
Total bilirubin, umol/L	7.4 (4.7–12.4)	7.5 (4.1–14.8)	6.5 (4.5–11.7)	8.2 (6.2–12.1)	0.400
Direct bilirubin, umol/L	3.6 (2.3–5.11)	2.6 (1.8–6.3)	3.5 (2.2–4.6)	4.3 (3.3–7.3)	0.450
Indirect bilirubin, umol/L	4.0 (2.1–6.8)	4.8 (2.1–8.3)	3.3 (1.7–6.1)	4.4 (2.7–5.0)	0.573
Blood urea nitrogen, mmol/L	3.8 (3.2–4.8)	4.2 (3.5–5.0)	3.6 (3.2–4.3)	3.5 (2.9–5.8)	0.120
Serum creatinine, umol/L	63.2 (51.8–75.6)	53.8 (49.1–73.2)	64.6 (53.9–77.1)	58.8 (50.6–82.3)	0.352
Phosphocreatine kinase, U/L	67.3 (51.4–101.4)	86.9 (57.5–104.5)	66.5 (45.1–91.2)	60.2 (43.9–108.4)	0.239
Lactate dehydrogenase >245 U/L—*n*/*N* (%)	40/69 (58.0)	6/16 (37.5)	25/43 (58.1)	9/10 (90.0)	0.026
Hydroxybutyrate dehydrogenase>182 U/L- *n*/*N* (%)	41/70 (58.6)	6/16 (37.5)	26/44 (59.1)	9/10 (90.0)	0.031
Potassium, mmol/L	3.9 (3.6–4.2)	3.9 (3.7–4.5)	3.8 (3.6–4.1)	4.1 (3.7–4.7)	0.350
Sodium, mmol/L	136.3 (134.8–138.2)	136.3 (135.7–137.9)	137.3 (134.9–138.6)	134.0 (131.0–135.9)	0.001
Erythrocyte sedimentation rate (*n* = 36)	19.0 (10.3–26.8)	15.0 (9.0–22.3)	19 (9.8–27.0)	22.0 (13.8–33.5)	0.626
C-reactive protein>5 mg/l—*n*/*N* (%)	27/67 (40.3)	1/14 (7.1)	18/43 (41.9)	8/10 (80.0)	<0.001
Abnormalities on chest CT—*n*/*N* (%)	52/69 (75.4)	0/15 (0.0)	42/44 (95.5)	10/10 (100.0)	<0.001
Ground-glass opacity	13/69 (18.8)	0/15 (0.0)	13/44 (29.5)	0/10 (0.0)	0.009
Local patchy shadowing	5/69 (7.2)	0/15 (0.0)	5/44 (11.4)	0/10 (0.0)	0.398
Bilateral patchy shadowing	30/69 (43.5)	0/15 (0.0)	24/44 (54.5)	6/10 (60.0)	<0.001
Interstitial abnormalities	4/69 (5.8)	0/15 (0.0)	0/44 (0.0)	4/10 (40.0)	<0.001

*Data are presented as medians (Interquartile ranges, IQR) and n/N (%), where N is the total number of patients with available data*.

*p-values comparing severe pneumonia and the other cases are from χ^2^ test, Fisher's exact test, or Kruskal-Wallis H test*.

**Figure 4:**
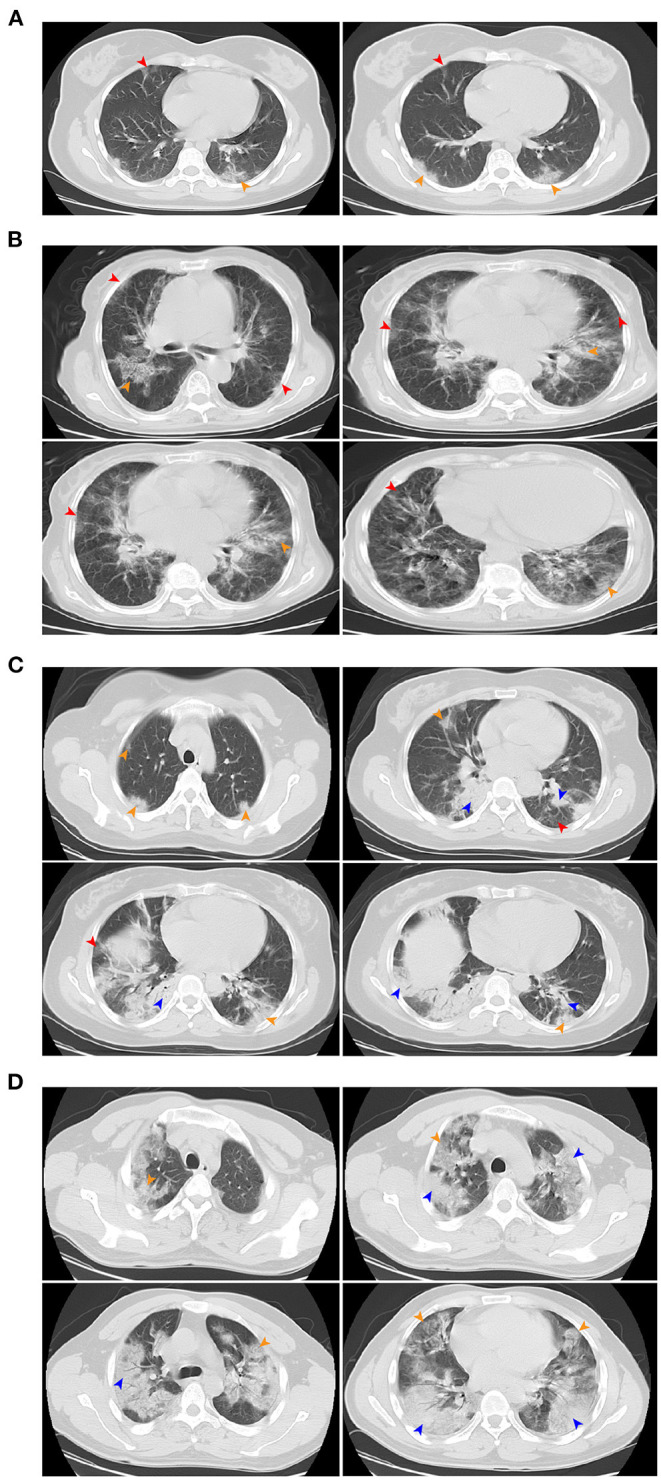
Chest CT images. **(A)** Chest CT images showing bilateral ground-glass opacity on day 5 after symptom onset. **(B)** Chest CT images showing bilateral ground-glass opacity on day 3 after symptom onset. **(C)** Chest CT images showing bilateral ground-glass opacity and bilateral subsegmental areas of consolidation on day 5 after symptom onset. **(D)** Chest CT images showing bilateral multiple lobular and subsegmental areas of consolidation on day 10 after symptom onset. **(A,B)** were mild pneumonia, **(C,D)** were severe pneumonia. Red arrow indicates ground-glass opacity, yellow arrow indicates nodular consolidation, blue arrow indicates the mixture areas of ground-glass opacity and nodular consolidation.

All patients except for one pregnant woman received a chest CT scan and most (52/69, 75.4%) showed evidence of pneumonia. The most common patterns on chest CT were bilateral patchy shadowing (43.5%) and ground-glass opacity (18.8%). Prominent radiologic abnormalities, including bilateral patchy shadowing, and interstitial abnormalities were more common among severe than non-severe cases (all *P* < 0.001). Figure 4 shows the representative radiologic findings of two non-severe cases and another two severe cases.

### Treatment and Outcomes

During hospital admission, complications were rare, and only one Tibetan patient (1.4%) with severe pneumonia developed ARDS. Fifty-seven (81.4%) patients received antiviral treatment, of whom 28.6 and 42.1% received empirical antibiotics and oseltamivir therapy, respectively. Severe cases were significantly more likely to receive treatment (all *P* < 0.05). Only five (7.1%) were given a systemic corticosteroid, of whom two had severe pneumonia. In addition, significantly more non-severe cases received traditional Chinese medicine than severe cases (83.3 and 40.0%, respectively; *P* < 0.05).

Oxygen therapy was initiated in 71.7 and 100% of non-severe cases and severe cases, respectively, and symptoms improved for most patients following a 4 L/minute oxygen inhalation using a nasal cannula. Only one severe case with ARDS received non-invasive mechanical ventilation for 10 min and was then switched to mask oxygen inhalation because of intolerance.

**Figure 5:**
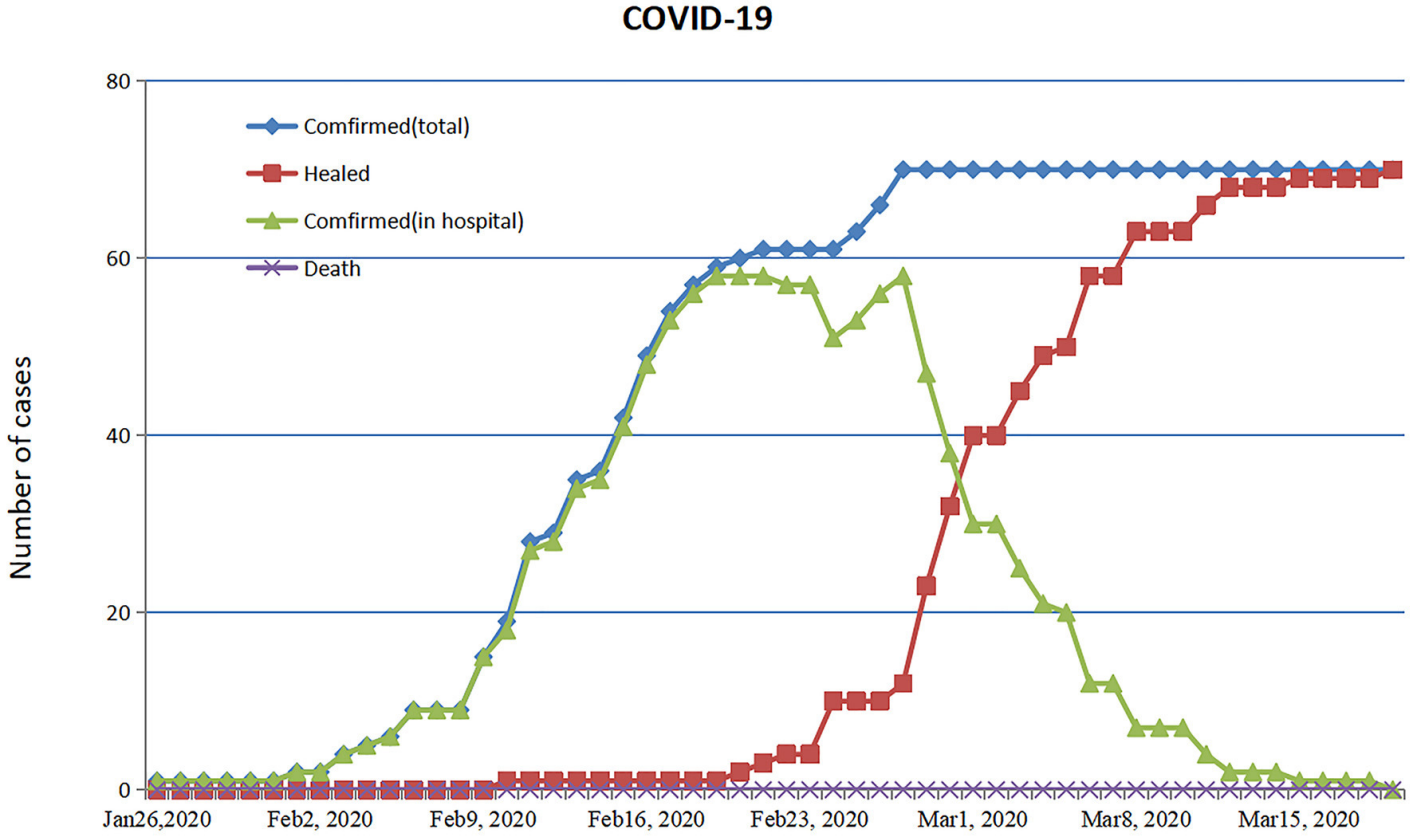
Timeline of treatment and clinical outcomes. Trend chart of confirmed cases of COVID-19 through 19 March 2020.

By 19 March, 2020, all 70 patients were discharged and no patients died (Figure 5). The patient with ARDS was moved to a designated hospital at a lower altitude (2,560 m) and improved significantly. Fitness for discharge was based on abatement of fever for at least 3 days, improved chest CT results, and viral clearance in samples from the upper respiratory tract. The median duration of a positive PCR result was 10 days, and severe cases had a significantly longer average duration of positive PCR than non-severe cases (Table 4).

**Table 4:** Treatment and outcomes of 70 patients with COVID-19.

**Characteristics**	**All patients (*n* = 70)**	**Disease severity**		***P*-value**
		**Non-severe case (*n* = 60)**	**Severe case (*n* = 10)**	
Duration from illness onset to admission-days	0 (0–1.0)	0 (0–1)	2.5 (2–10)	<0.001
Duration of SARS-CoV-2 RNA positive	10 (8–13)	10 (8–12)	14 (11–16)	0.002
**Treatment—*****n*** **(%)**				
Antiviral therapy	57 (81.4)	47 (78.3)	10 (100.0)	0.190
Administration of oseltamivir	24 (42.1)	16 (26.7)	8 (80.0)	0.002
Antibiotic therapy	20 (28.6)	11 (18.3)	9 (90.0)	<0.001
Use of corticosteroid	5 (7.1)	3 (5.0)	2 (20.0)	0.146
Traditional Chinese medicine	54 (77.1)	50 (83.3)	4 (40.0)	0.007
Oxygen support—*n* (%)	53 (75.7)	43 (71.7)	10 (100.0)	0.104
**Complications—*****n*** **(%)**				
Acute respiratory distress syndrome	1 (1.4)	0 (0.0)	1 (10.0)	0.143
**Prognosis—*****n*** **(%)**				
Hospitalization	30 (42.9)	37 (61.7)	3 (30.0)	0.087
Discharge	40 (57.1)	23 (38.3)	7 (70.0)	–

*Data are presented as medians (interquartile ranges, IQR) and n (%)*.

*p-values comparing severe pneumonia and the other cases are from χ^2^ test, Fisher's exact test, or Mann-Whitney U test*.

*Non-severe case including uncomplicated illness and mild pneumonia*.

## Discussion

SARS-CoV-2 utilizes the angiotensin-converting enzyme 2 (ACE2) receptor to enter cells and it is postulated that the human body is more vulnerable to infectious diseases like COVID-19 during harsh winters (21, 22). This study reports on an outbreak of COVID-19 in the plateau region of Western China during a particularly cold winter. More than half of the patients were male and included both young children and older individuals >65 years of age. None of the infected patients had a previous history of contact with people from Wuhan, and 60.0% (42/70) of patients were associated with clusters, of whom 20.0% (12/70) were linked to familial clusters, a finding consistent with previous reports (7, 20, 23–25). These results confirm that the mass gatherings increased the risk of SARS-CoV-2 infection.

In the early phase of the COVID-19 outbreak in Wuhan, human-to-human transmission occurred among some cases, leading to subsequent community outbreaks before control measures could be implemented. As a result, the COVID-19 fatality rate was higher in Wuhan than in other regions (7). In the current study, the patients from Daofu had more mild symptoms than patients associated with early COVID-19 outbreaks in Wuhan (20). This may be because most COVID-19 patients in Daofu (58/70, 82.9%) were found by active screening. Indeed, the incidence of severe pneumonia, fever, dyspnea, and interstitial abnormalities observed by chest CT were all significantly lower among screened patients than those admitted after symptom onset (all *P* < 0.05). Thus, the implementation of active screening as a prevention strategy resulted in early diagnosis and better disease management, contributing to a lower incidence of severe pneumonia in the hypoxic plateau region of Sichuan. Twenty patients without symptoms were also diagnosed by active screening in this study. SARS-CoV-2 transmission from asymptomatic individuals, or those still within the incubation period, was described previously (26–29). As a result, the potential widespread transmission of SARS-CoV-2 could not be prevented without the active screening of asymptomatic populations. In addition, viral loads measured by qRT-PCR are, at best, a crude measure of how much infectious virus is being shed, so further research is needed to quantify viral loads in both asymptomatic and pre-symptomatic cases (30).

Fever is the most common symptom of COVID-19 in prior studies (7, 8, 20). However, in the current study fever only occurred in 12.9% of the patients. Fever was less predominant in the hypoxic plateau region of Sichuan than in other areas of China. This finding indicates that many COVID-19 patients could be missed if the surveillance case definition required fever detection. In addition, it is reported that some COVID-19 patients develop ARDS or multiple organ failure resulting from a cytokine storm (31). In this study, only 12.9 and 1.4% of patients developed dyspnea and ARDS, respectively. Most patients with severe pneumonia had no serious symptoms. These results are similar to those seen in high altitude regions of Bolivia, central Ecuador, and Nepal (32–34). There a several possible explanations for the low rates of COVID-19 transmission and disease severity in these regions: (1) individuals living in high plateau regions are more adapted to a hypoxic environment and thus develop less serious disease (13, 35, 36); (2) reduced air pollution and increased vitamin D levels at high altitude result in milder symptoms (37, 38); (3) whether individuals in the plateau of Sichuan have ACE2 gene variants as reported (39, 40). Each of these theories will require additional investigation. Importantly, COVID-19 patients in high altitude plateau areas have relatively mild and atypical symptoms that could delay testing and increase the risk of epidemic spread without the use of active screening.

Testing resources are limited in the hypoxic plateau areas of Sichuan because of poor medical conditions. Consistent with other reports, lymphopenia was common in the COVID-19 patients in this study (20). While elevated aminotransferase levels were also found in 45.7% of patients, hepatic abnormalities were mild to moderate, and most patients recovered. No apparent radiologic manifestations were noted in more than 20.0% of laboratory-confirmed symptomatic cases. These findings indicated that some patients had isolated SARS-CoV-2 infection before or without the development of viral pneumonia, emphasizing the importance of identifying and managing them before the disease progressed.

Most patients in this study received antiviral treatment, but the specific treatment varied among patients. Less than one-third received antibiotics and only a few patients were treated with steroids for 3–5 days. It is unclear whether the use of antivirals, antibiotics, or steroids affected patient prognosis. Eighty percent of the patients with severe pneumonia had at least one underlying disorder such as hypertension, diabetes, or chronic obstructive pulmonary disease. Symptoms of severe pneumonia subsided within a few days and pulmonary lesions gradually decreased after timely treatment of any underlying conditions or transport to a lower altitude hospital when needed.

There were some limitations to the present study. First, limited tracking and tracing capacity in high-altitude regions may allow a higher proportion of asymptomatic cases to go undetected. However, this would further support the argument for reduced case severity at high altitudes. Second, the retrospective study design and limited number of participants may have introduced bias. Third, because most of the patient treatment courses were descriptive, this did not allow for a detailed calculation and statistical comparison.

In conclusion, mass gatherings increased risk of spread of SARS-CoV-2 responsible for COVID-19. Active screening and early management have collectively contributed to reduced incidence of severe pneumonia and satisfactory prognoses of infections with COVID-19 in the plateau region of Sichuan. Due to a lack of medical resources in this plateau region, many tests, including those that assess immune function or test for inflammatory indicators, could not be carried out, resulting in incomplete data. However, the present study could provide real-world experience for control strategies of COVID-19 in remote plateau regions.

## Data Availability Statement

The raw data supporting the conclusions of this article will be made available by the authors, without undue reservation.

## Ethics Statement

The studies involving human participants were reviewed and approved by the Institutional Review Board Committee of West China Hospital of Sichuan University. The patients/participants provided their written informed consent to participate in this study.

## Author Contributions

G-pL, HT, and J-jL conceptualized the paper. G-gJ collected and analyzed the data, with input from K-jX, HY, RS, YC, and T-yY. G-pL, HT, J-jL, G-gJ, and XH wrote the initial draft with all authors providing critical feedback and edits to subsequent revisions. HT and G-pL attests that all listed authors meet authorship criteria and that no others meeting the criteria have been omitted. All authors approved the final draft of the manuscript.

## Funding

This work was supported by 2019-nCoV tackling project of Chengdu Science and Technology Bureau (2020-YF05-00003-SN).

## Conflict of Interest

The authors declare that the research was conducted in the absence of any commercial or financial relationships that could be construed as a potential conflict of interest.

## Publisher's Note

All claims expressed in this article are solely those of the authors and do not necessarily represent those of their affiliated organizations, or those of the publisher, the editors and the reviewers. Any product that may be evaluated in this article, or claim that may be made by its manufacturer, is not guaranteed or endorsed by the publisher.
